# Systematic control of α-Fe_2_O_3_ crystal growth direction for improved electrochemical performance of lithium-ion battery anodes

**DOI:** 10.3762/bjnano.8.204

**Published:** 2017-09-28

**Authors:** Nan Shen, Miriam Keppeler, Barbara Stiaszny, Holger Hain, Filippo Maglia, Madhavi Srinivasan

**Affiliations:** 1 BMW-NTU Future Mobility Research Lab, Nanyang Technological University, School of Materials Science and Engineering, and Energy Research Institute at Nanyang (ERI@N), Research Techno Plaza, X-Frontier Blk, 50 Nanyang Drive, Singapore 637553, Singapore,; 2 BMW Group, Petuelring 130, 80788 Munich, Germany

**Keywords:** 2,3-diaminobutane, 1,2-diaminopropane, ethylenediamine, lithium-ion battery, shape-controlled synthesis

## Abstract

α-Fe_2_O_3_ nanomaterials with an elongated nanorod morphology exhibiting superior electrochemical performance were obtained through hydrothermal synthesis assisted by diamine derivatives as shape-controlling agents (SCAs) for application as anodes in lithium-ion batteries (LIBs). The physicochemical characteristics were investigated via XRD and FESEM, revealing well-crystallized α-Fe_2_O_3_ with adjustable nanorod lengths between 240 and 400 nm and aspect ratios in the range from 2.6 to 5.7. The electrochemical performance was evaluated by cyclic voltammetry and charge–discharge measurements. A SCA test series, including ethylenediamine, 1,2-diaminopropane, 2,3-diaminobutane, and *N*-methylethylenediamine, was implemented in terms of the impact on the nanorod aspect ratio. Varied substituents on the vicinal diamine structure were examined towards an optimized reaction center in terms of electron density and steric hindrance. Possible interaction mechanisms of the diamine derivatives with ferric species and the correlation between the aspect ratio and electrochemical performance are discussed. Intermediate-sized α-Fe_2_O_3_ nanorods with length/aspect ratios of ≈240 nm/≈2.6 and ≈280 nm/≈3.0 were found to have excellent electrochemical characteristics with reversible discharge capacities of 1086 and 1072 mAh g^−1^ at 0.1 C after 50 cycles.

## Introduction

Since conventional transportation is seen as problematic in terms of fossil fuel consumption and human-induced greenhouse gas emissions [1], battery electric vehicles (BEVs) have moved into the focus of the automotive industry. As power sources, lithium-ion batteries (LIBs) are considered as the most promising candidates, since LIBs offer the highest energy density of all known rechargeable battery systems [2–4]. In order to address today’s challenges of electromobility (e.g., customer acceptance of BEVs by extending driving ranges, faster charging, and lower costs), the development and optimization of electrode materials are of great interest. Considering that a target driving range of 300 miles is required for BEVs to achieve a sustainable mass market penetration (as defined by the US Department of Energy), the energy density of the BEV power source must be increased by a factor of 2.5 by 2030 [5]. Since the commercialization of LIBs in 1991 by the Sony Cooperation [6–7], carbon-based materials such as graphite have predominantly been applied as negative electrodes. Although graphite is advantageous due to its high coulombic efficiency, its flat voltage curve and the low operating voltage of 0.1 V vs Li/Li^+^ it is limited to a lithium storage capacity of only 372 mAh g^−1^, given a stoichiometry of LiC_6_ [8]. In 2000, the Tarascon research group brought attention to transition metal oxides as a new class of possible anode materials that showed capacities in the range of twice as high as graphite [9], and some of them even show values higher than 1000 mAh g^−1^ with the interaction with lithium ions [10]. Therefore, transition metal oxides are candidates as new, high-capacity, electrode active materials in next generation LIBs. Among them, α-Fe_2_O_3_ (hematite) has attracted more and more attention due to its non-toxicity, high corrosion resistance, low processing costs, and especially, for its high theoretical gravimetric lithium-ion storage capacity of 1007 mAh g^−1^. However, α-Fe_2_O_3_ interacts with lithium ions via a conversion reaction, leading to finely dispersed metal nanocrystals in a Li_2_O matrix. This process is associated with large volume changes and also exhibits irreversible phase transformation (from a hexagonal anionic packing to a cubic phase [11]) that causes a poor long-time cycling behavior with severe capacity fading [12–14]. Possibilities to solve these problems include shape controlling towards nanometer-scale morphologies. It has been widely demonstrated that nanometer-scale α-Fe_2_O_3_ particles show excellent electrochemical performance in the reaction with lithium ions as compared to micrometer-scale crystals. This can be attributed to the comparatively shorter lithium-ion path ways and better ability to accommodate the strain during volume change caused by lithiation/delithiation [15]. The delithiation step, which is essential for the reaction reversibility, is considered thermodynamically impossible for micrometer-sized materials, but becomes feasible for their nanostructured derivatives [9,11,16–20]. Therefore, morphology control and nanostructuring of α-Fe_2_O_3_ is of great topical importance. Among several synthesis methods for α-Fe_2_O_3_ crystal phases, including precipitation and sol−gel approaches, the hydro/solvothermal synthesis route is known to be advantageous due to the homogenous nucleation/growth process and highly crystallized products [20–22]. In order to develop a synthesis method with morphology control aspects, inorganic ions as well as organic reagents (including phosphates, sulfates, chlorides, fluorides, benzenediols, nitrilotriacetic acid and diamine derivatives) have been successfully applied as shape-controlling agents (SCAs) to obtain α-Fe_2_O_3_ with various morphologies enclosed by different crystal facets [22–27]. The diamine-assisted hydrothermal synthesis of α-Fe_2_O_3_ nanoparticles is seen as beneficial, being a low-cost and time-efficient combination of an efficient SCA and a powerful synthesis tool. The resulting α-Fe_2_O_3_ is free from nitrogen (elemental analysis) [28], since the applied diamine derivatives are normally water-soluble and can be easily removed. With this technique, expensive, difficult to scale-up, structure-directing approaches involving sacrificial hard templates can be avoided. The aspect ratio of the resulting nanorod morphology is adjustable by the type of diamine derivative used and other reaction parameters. Li et al. applied a hydrothermal synthesis assisted by 1,2-diaminopropane as an SCA and obtained crystallized α-Fe_2_O_3_ nanorods with high and controllable aspect ratios [22]. Lin et al. demonstrated that such α-Fe_2_O_3_ nanorods outperform sub-micrometer and micrometer-sized particles in terms of electrochemical performance in LIBs [17]. Also, ethylenediamine is used in the hydrothermal synthesis of α-Fe_2_O_3_ nanoparticles, leading to a shuttle-like nanorod morphology [27]. Diamines increase the pH of the reaction mixture, supporting the phase transformation of FeOOH to α-Fe_2_O_3_. In addition, diamines form chelate complexes with ferric ions in solution, leading to a directional dependency of OH^−^ attacks for subsequent hydrolysis processes, which results in a targeted growth direction of iron oxide products [29]. Furthermore, diamines directly coordinate to ferric lattice species and the complexes are released into solution. Since it is understood that this coordination will specifically take place on the α-Fe_2_O_3_ surface facets with only singly coordinated hydroxyl groups, this interaction mechanism can be applied to direct the crystal growth in a specific growth direction. It was found that 1,2-diaminopropane outperforms ethylenediamine as an SCA. Also, the vicinal position of the two amine groups seems to be crucial for shape controlling, since it was reported that 1,3-diaminopropane shows little effect on directing the crystal growth of α-Fe_2_O_3_. [22]. To gain a deeper understanding of the role of diamines in the shape-controlled synthesis of α-Fe_2_O_3_, a detailed test series, including ethylenediamine, 1,2-diaminopropane, 2,3-diaminobutane and *N*-methylethylenediamine, is presented. The effect of the increasing number of H_3_C-groups, contributing to a larger steric hindrance and an increase in the electron density (+M effect) at the reactive diamine unit, is investigated. *N*-methylethylenediamine was included since it is a constitutional isomer to 1,2-diaminopropane and allows investigation in terms of the position of the H_3_C-group. To the best of our knowledge, 2,3-diaminobutane and *N*-methylethylenediamine are presented for the first time as SCAs in α-Fe_2_O_3_ synthesis.

## Experimental

In a typical procedure, 32 mL of 1,2-diaminopropane (99%, Sigma-Aldrich) was added drop wise into 18 mL of 1.5 M FeCl_3_·6H_2_O (99%, Riedel-de Haën) under constant magnetic stirring. Subsequently, the solution was transferred to a 100 mL autoclave with teflon-lined stainless steel for hydrothermal treatment at 180 °C for 16 h. The product was allowed to naturally cool down to room temperature after reaction and collected by centrifugation with deionized water and ethanol. The sample was dried overnight at 60 °C, and a red-colored product was obtained. For a systematic study, the concentration of FeCl_3_·6H_2_O was varied, while the ratio between iron ions and organic SCA was kept constant. The effectiveness of the SCA was compared by using ethylenediamine (99%, Sigma-Aldrich), 2,3-diaminobutane (95%, Otava) and *N*-methylethylenediamine (95%, Sigma-Aldrich) instead of 1,2-diaminopropane.

X-ray diffraction (XRD) analysis was performed on a Shimadzu XRD-6000 diffractometer operating at 40 kV and 40 mA using Cu Kα radiation (λ = 0.154 nm) with a copper target and a nickel filter. The surface morphology and microstructure of the samples was analyzed using a JEOL 6340F field emission scanning electron microscope (FESEM) in secondary electron imaging mode. The accelerating voltage was set to 5 kV.

The electrodes were prepared by mixing 40% of as-synthesized active iron oxide powder with 40% of conductive additives (Super P Li carbon, Timcal) and 20% of polyvinylidene difluoride (PVdF) binder (Kynar 2801) by weight in 1-methyl-2-pyrrolidinone (NMP, 99.5%, Sigma-Aldrich). This was coated on a copper foil with a doctor blade (gap height of 300 μm) then oven-dried at 60 °C overnight and roll-pressed with 20 tons. The dried electrode had a layer thickness of around 30 μm and a mass loading of around 2.5 mg cm^−2^. The porosity of the electrode was estimated to be around 30% by calculating the ratio between theoretical volume and the real volume of the electrode. The electrode was assembled into a coin cell (CR2016) with lithium foil (0.59 mm thickness, Hohsen Corp., Japan) as both a counter and a reference electrode in an argon-filled glove box (H_2_O, O_2_ < 1 ppm, Mbraun, Unilab, USA). 150 μL of LiPF_6_ (1 M) in ethylene carbonate/diethyl carbonate (1:1 w/w, Danvec) was used as the electrolyte and a Celgard 2400 membrane was used as the separator. The cycling performance tests were conducted with a multichannel battery tester (Neware Technology Limited) with discharge and charge current density of 0.1 C (100 mAh g^−1^) at room temperature. Rate performance tests were conducted using the same instrument with charge current of 0.1 C and discharge current varying from 0.1 to 3 C. Cycling voltammetry (CV) was performed in the range of 0.05 to 3.00 V with a sweep rate of 0.1 mV s^−1^ on a Solartron electrochemical workstation (1470E and SI 1255B impedance/gain-phase analyzer coupled with a potentiostat).

## Results and Discussion

### Structural characterization

**Figure 1:**
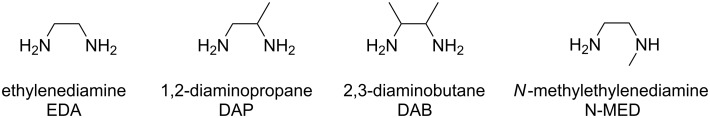
Schematic illustration of applied SCAs containing two vicinal amine groups and different H_3_C-groups.

The sample nomenclature was set to α-Fe_2_O_3_-SX.X, in which S refers to the type of SCA (E for ethylenediamine, D for 1,2-diaminopropane, B for 2,3-diaminobutane, and N for *N*-methylethylenediamine) and X.X refers to the amount of Fe^3+^ in moles, which was varied over a range of 0.1 to 2.0. The hydrothermal synthesis parameters of all samples were set to a 1:13 molar ratio of FeCl_3_/SCA and the reaction temperature/time was 180 °C/16 h, which was optimized towards fully crystalized pure phase products in a preliminary test series and were found to be in good agreement with previously reported α-Fe_2_O_3_ synthesis [22,30]. A systematic study was conducted to analyze the effect of Fe^3+^ concentration and various SCAs on crystal phase formation and morphology of α-Fe_2_O_3_. All applied diamine derivatives are schematically illustrated in Figure 1.

For comparison, approaches without an SCA but using NaOH_(aq)_ to adjust the pH value for the phase transformation of FeOOH to α-Fe_2_O_3_ were also conducted. These approaches result in micrometer-sized particles without a periodically repeating unit in the nanometer regime.

**Figure 2:**
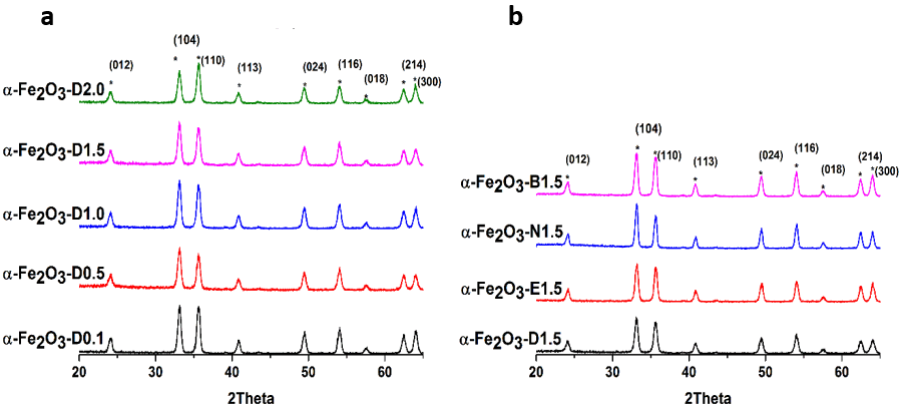
a) XRD patterns of α-Fe_2_O_3_ nanoparticles synthesized with various Fe^3+^ concentrations and 1,2-diaminopropane as the SCA and b) different diamine derivatives as SCAs and a fixed Fe^3+^ concentration.

Figure 2a illustrates the XRD patterns of samples obtained from a test series with 1,2-diaminopropane as the SCA and a varied Fe^3+^ concentration (α-Fe_2_O_3_-D0.1, α-Fe_2_O_3_-D0.5, α-Fe_2_O_3_-D1.0, α-Fe_2_O_3_-D1.5, and α-Fe_2_O_3_-D2.0), whereas Figure 2b shows the diffractograms of samples obtained from a test series with a constant Fe^3+^ concentration (here 1.5 M) and varied SCA (α-Fe_2_O_3_-E1.5, α-Fe_2_O_3_-D1.5, α-Fe_2_O_3_-B1.5 and α-Fe_2_O_3_-N1.5).

The reflections reported in Figure 2 indicate well-crystalized materials that can be indexed to α-Fe_2_O_3_ (JAPDS card No. 330664). The lattice constants were calculated using Rietveld refinement and found to be in the range of 5.030 to 5.033 Å for lattice constant *a*, and 13.748 to 13.757 Å for lattice constant *c* (see Table 1), which is in perfect agreement with reported values [31–32]. The obtained *R*_Bragg_ values vary from ≈3% to ≈7%, indicating a good reliability of the refined data. No reflections of detectable impurities were observed, indicating that diamines are useful to control the crystal growth direction, but do not have any observable impact on the crystal phase formation.

**Table 1:** Physicochemical characteristics of α-Fe_2_O_3_ synthesized with different SCAs and various Fe^3+^ concentrations. DAP: 1,2-diaminopropane, EDA: ethylenediamine, DAB: 2,3-diaminobutane, N-MED: *N*-methylethylenediamine.

Test series	Sample ID	Fe^3+^ conc. (M)/SCA	Lattice constant, *a* (Å)	Lattice constant, *c* (Å)	*R*_Bragg_ value (%)	Rod length (nm)	Aspect ratio^a^

Varied Fe^3+^ conc., fixed SCA (EDA)	α-Fe_2_O_3_-E0.1	0.1/EDA	5.031	13.752	6.406	spherical particle	–
	α-Fe_2_O_3_-E0.5	0.5/EDA	5.033	13.753	5.158	spherical particle	–
	α-Fe_2_O_3_-E0.8	0.8/EDA	5.033	13.757	4.838	275	2.9
	α-Fe_2_O_3_-E1.0	1.0/EDA	5.033	13.755	5.184	280	3.0
	α-Fe_2_O_3_-E1.5	1.5/EDA	5.033	13.757	6.672	276	3.0
	α-Fe_2_O_3_-E2.0	2.0/EDA	5.030	13.749	5.819	277	2.9
Varied Fe^3+^ conc., fixed SCA (DAP)	α-Fe_2_O_3_-D0.1	0.1/DAP	5.031	13.750	4.636	spherical particle	–
	α-Fe_2_O_3_-D0.5	0.5/DAP	5.031	13.748	5.209	237	2.6
	α-Fe_2_O_3_-D0.8	0.8/DAP	5.030	13.749	2.997	320	4.7
	α-Fe_2_O_3_-D1.0	1.0/DAP	5.030	13.753	4.496	397	5.7
	α-Fe_2_O_3_-D1.5	1.5/DAP	5.031	13.750	3.645	394	5.0
	α-Fe_2_O_3_-D2.0	2.0/DAP	5.032	13.754	4.467	399	4.9
Varied SCA, fixed Fe^3+^ conc. (0.5M)	α-Fe_2_O_3_-E0.5	0.5/EDA	5.033	13.753	5.158	spherical particle	–
	α-Fe_2_O_3_-D0.5	0.5/DAP	5.031	13.748	5.209	237	2.6
	α-Fe_2_O_3_-B0.5	0.5/DAB	5.033	13.750	7.067	particle	–
	α-Fe_2_O_3_-N0.5	0.5/N-MED	^b^	^b^	^b^	^b^	^b^
Varied SCA, fixed Fe^3+^ conc. (1.5M)	α-Fe_2_O_3_-E1.5	1.5/EDA	5.033	13.757	6.672	276	3.0
	α-Fe_2_O_3_-D1.5	1.5/DAP	5.031	13.750	3.645	394	5.0
	α-Fe_2_O_3_-B1.5	1.5/DAB	5.032	13.751	5.073	256	2.8
	α-Fe_2_O_3_-N1.5	1.5/N-MED	5.031	13.748	7.019	spherical particle	–

^a^Aspect ratio is not calculated for samples without nanorod morphology. ^b^Not synthesized, since an even higher Fe^3+^ concentration of 1.5 M did not lead to a nanorod morphology.

**Figure 3:**
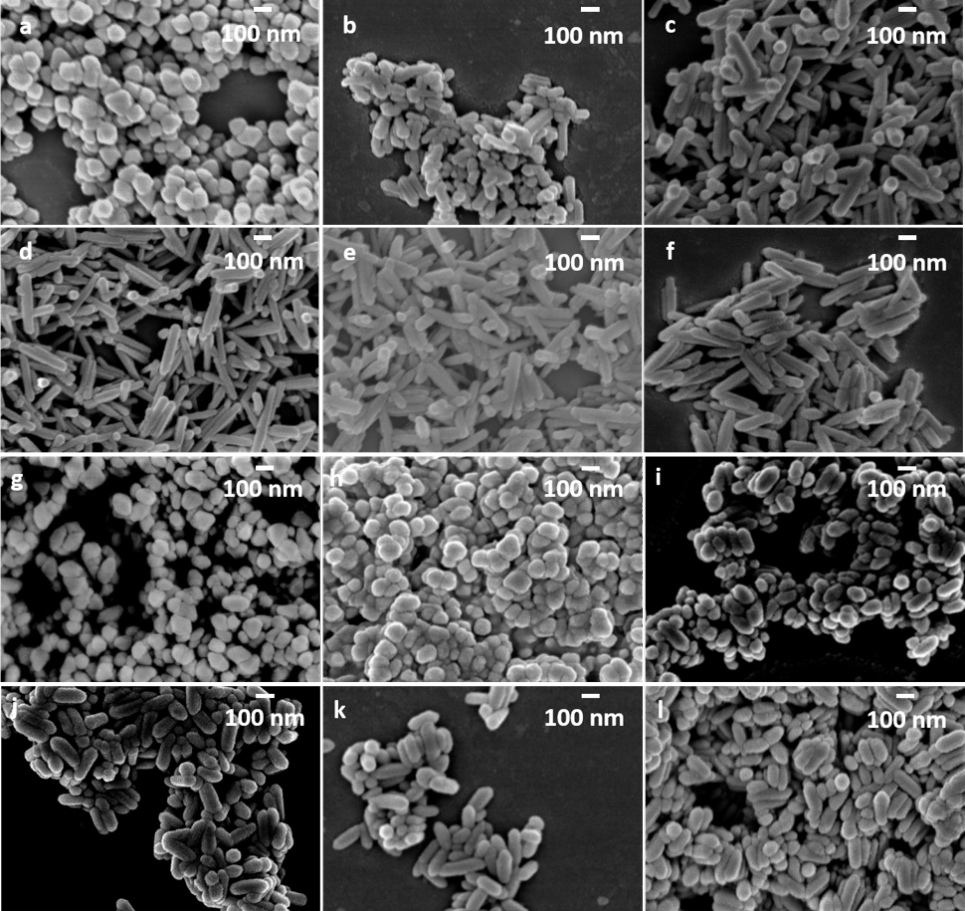
FESEM images of α-Fe_2_O_3_ nanoparticles obtained with 1,2-diaminopropane as the SCA and varied Fe^3+^ concentration of a) 0.1 M, b) 0.5 M, c) 0.8 M, d) 1.0 M, e) 1.5 M and f) 2.0 M; and α-Fe_2_O_3_ nanoparticles obtained with ethylenediamine as the SCA and varied Fe^3+^ concentration of g) 0.1 M, h) 0.5 M, i) 0.8 M, j) 1.0 M, k) 1.5 M and l) 2.0 M; magnification: 50,000.

FESEM was applied to examine the morphology of the final α-Fe_2_O_3_ products. Figure 3a–f displays the morphology evolution with increased Fe^3+^ concentration from 0.1 to 2.0 M and 1,2-diaminopropane used as an SCA. At an Fe^3+^ concentration of 0.1 M, nanoparticles with a diameter of ≈100 nm are obtained. When the Fe^3+^ concentration is increased to 0.5 M, a 1-D rod-like morphology starts to form with rod length of ≈240 nm, resulting in an aspect ratio of ≈2.6. Increasing the Fe^3+^ concentration to 0.8 M leads to an elongated nanorod shape with rod length of ≈320 nm and an aspect ratio of ≈4.7. At higher Fe^3+^ concentrations from 1.0 to 2.0 M, pronounced elongated nanorods with a length of ≈400 nm and aspect ratio in the range of 4.9 to 5.7 were obtained.

**Figure 4:**
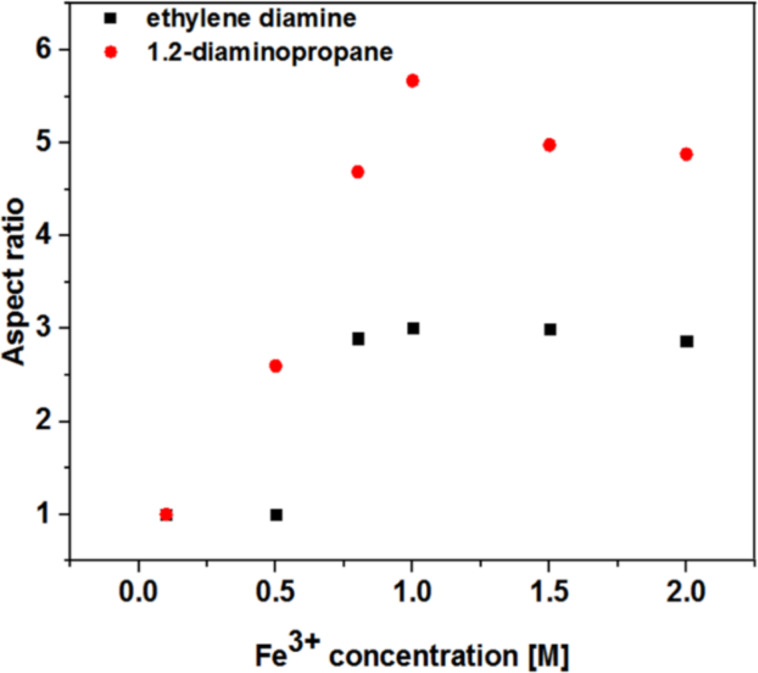
Aspect ratio vs Fe^3+^ concentration for α-Fe_2_O_3_ nanoparticles obtained with 1,2-diaminopropane and ethylenediamine as the SCA (the aspect ratio for spherical particles is set to 1; 20 nanorods were taken for the estimation of the aspect ratio for each Fe^3+^ concentration; a certain fluctuation margin cannot be excluded especially due to a natural variation in the rod length of about 20%).

To verify the impact of the additional H_3_C-group at the vicinal diamine unit, 1,2-diaminopropane is substituted by ethylenediamine, while all further reaction parameters are kept constant. In general, the Fe^3+^ concentration vs aspect ratio dependence shows an analogical profile shape as obtained for 1,2-diaminopropane, but shorter rod length for each tested Fe^3+^ concentration (Figure 3g–l and Figure 4). In detail, at lower Fe^3+^ concentrations of 0.1 M and 0.5 M, nanoparticles with a diameter of ≈100 nm are obtained with ethylenediamine as the SCA. When the Fe^3+^ concentration is increased to 0.8 M, nanorods with a length of ≈275 nm and aspect ratio of ≈2.9 are achieved. A further increase of the Fe^3+^ concentration up to 2.0 M leads to a comparable rod-like morphology with a rod length of ≈280 nm and aspect ratio of ≈3.0.

Diamines have several substantial effects in the α-Fe_2_O_3_ synthesis that in general involves a two-step phase transformation, formally described as Fe(OH)_3_ → FeOOH → α-Fe_2_O_3_. The p*K*_b_ is decreased in the presence of diamines, which accelerates the conversion of FeOOH to α-Fe_2_O_3_ [28]. In addition, the crystal growth direction of iron oxide polymorphs like FeOOH, Fe_2_O_3_ or Fe_3_O_4_ is controlled by diamines. Among other synthesis parameters (concentration, reaction time, temperature and solvents) [27,29,33], the shape controlling ability can be adjusted by substituents located at the reactive diamine unit that affects electronic density (+M effect) and steric interactions, which is verified by results of our test series. Diamines are typical chelate ligands that interact with appropriate coordination centers. First, octahedral-shaped complexes are formed [29], formally described as (Fe[C_2_H_2_N_2_R^1^R^2^]_3_)^3+^. Diamine derivatives, in which the two amine groups are located at adjacent C atoms (vicinal position) are assumed to be supportive, since a thermodynamically favored 5-membered ring can be formed. This possibly explains the superior shape-controlling ability of 1,2-diaminopropane over 1,3-diaminopropane. Besides shape control, the complex formation has a second effect: the free-iron concentration in the solution is reduced, which separates nucleation and growth processes and supports high quality crystallization [27].

The complex stability is expected to decrease during synthesis with increasing temperature and pressure until the Fe^3+^ in complex formation can be successfully attacked by OH^−^. This will result in the gradual loss of diamine ligands, leading to complexes with two OH^−^ groups and two diamines that form a quasi-square-plane perpendicular to the OH^−^ direction [29]. Therefore, the following hydrolysis process is inhibited in the planar direction, but favored in the normal direction, supporting the growth of FeOOH along the [110] direction [22,29]. In addition, the growth direction of α-Fe_2_O_3_ is dictated since diamines not only interact with free-iron ions in solution, but also coordinate directly to the ferric lattice, and the complexes are released into solution [28]. The diamine adsorption capacity of α-Fe_2_O_3_ surfaces varies for differently indexed crystal facets depending on their loading with singly, doubly, triply or geminal coordinated hydroxyl groups [34]. Only singly coordinated hydroxyl groups are assumed to be involved in the interaction with the diamines [35]. According to Barrón et al., singly coordinated hydroxyl groups are located at the [100], [110], [012], [104] and [113] facets of natural and artificial α-Fe_2_O_3_ [36]. Hence the crystal growth in these directions is minimized, leading to α-Fe_2_O_3_ products with elongated shapes along the [001] direction.

**Figure 5:**
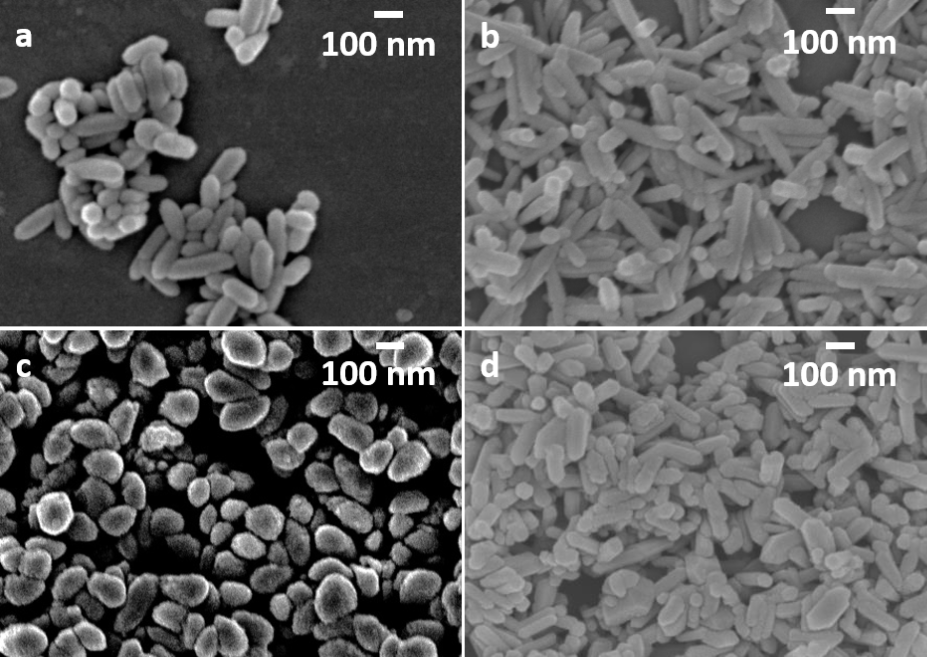
FESEM images of α-Fe_2_O_3_ nanoparticles obtained with a) ethylenediamine, b) 1,2-diaminopropane, c) *N*-methylethylenediamine and d) 2,3-diaminobutane, where the Fe^3+^ concentration was kept at 1.5 M, magnification: 50,000.

All supposed reaction mechanisms involve the direct interaction with ferric species and the freely accessible diamine unit. To support this assumption, *N*-methylethylenediamine was also tested as an SCA, since it is a constitutional isomer to 1,2-diaminopropane and allows investigation in terms of the position of the H_3_C-group. Consistent with other tested diamines, *N*-methylethylenediamine leads to fully crystallized α-Fe_2_O_3_ products (Figure 2). However, even when following these shape-controlling concepts, *N*-methylethylenediamine-assisted hydrothermal approaches did not lead to a nanorod morphology, but rather spherically shaped particles with a diameter of ≈100 nm (Fe^3+^ conc.: 1.5 M, Figure 5), indicating that *N*-methylethylenediamine substantially supports the α-Fe_2_O_3_ synthesis by providing an appropriate alkaline reaction medium for the phase transformation of FeOOH to α-Fe_2_O_3_. It can be concluded that the H_3_C-shift from C to N suppresses the ferric complex formation due to steric hindrance, and therefore, the directional dependency of the crystal growth is inhibited.

Since 1,2-diaminopropane shows better shape-controlling ability compared to ethylenediamine (possibly attributed to the additional H_3_C-group that supports complex formation by increased electron density (+M effect)), 2,3-diaminobutane was applied to investigate the impact of a second H_3_C-group. Besides an increased electron density, the steric hindrance caused by the exchange of H with the comparative bulky H_3_C-group (especially for the direct interaction with surface lattice ferric species in the later formation process) needs to be investigated. Fully crystallized α-Fe_2_O_3_ nanorods were obtained with 2,3-diaminobutane with a length of ≈260 nm and aspect ratio of ≈2.8 (Fe^3+^ conc.: 1.5 M). As discussed above, equivalent synthesis conditions led to slightly longer nanorods of ≈280 nm with an aspect ratio of ≈3.0 when ethylenediamine was used as the SCA. The use of 1,2-diaminopropane leads to products with longer lengths of ≈390 nm and aspect ratios of ≈5.0, indicating a marginal negative impact on the second H_3_C group on the complex formation with Fe^3+^ ions in solution and/or the interaction with the ferric species of the α-Fe_2_O_3_ surface due to steric hindrance. In general, the shape-controlling ability decreases in the following order: 1,2-diaminopropane > ethylenediamine ≥ 2,3-diaminobutane >> *N*-methylethylenediamine, which is in perfect agreement with the electronic and steric conditions of the applied diamines with respect to the above mentioned Fe-diamine interaction concepts.

**Figure 6:**
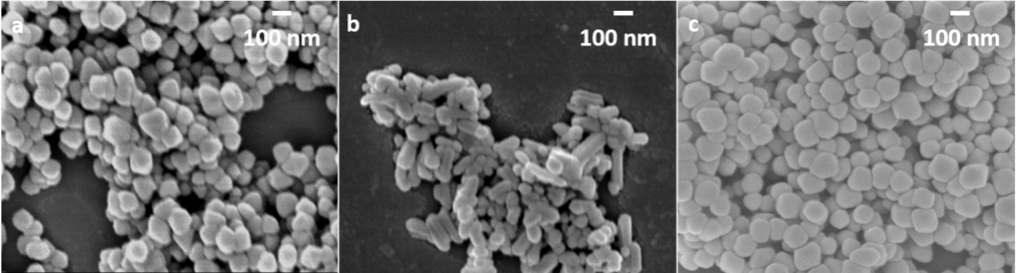
FESEM images of α-Fe_2_O_3_ nanoparticles obtained with a) ethylenediamine, b) 1,2-diaminopropane and c) 2,3-diaminobutane, where the Fe^3+^ concentration was kept at 0.5 M, magnification: 50,000.

The improved shape-controlling ability of 1,2-diaminopropane is also confirmed in the Fe^3+^ concentration test series. For 1,2-diaminopropane-assisted synthesis, the nanorod formation can already be observed with comparatively low Fe^3+^ concentrations of 0.5 M leading to products with ≈240 nm in length and aspect ratios of ≈2.6. The application of the other discussed diamine derivatives results in spherical particles of ≈100 nm diameter under the same conditions (Figure 6).

### Electrochemical studies

The interaction of lithium with α-Fe_2_O_3_ is assumed to occur via a multistep reaction with a conversion mechanism according to the following equations [37]:

[1]
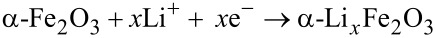


[2]
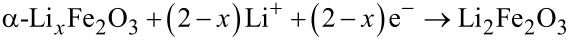


[3]
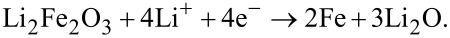


**Figure 7:**
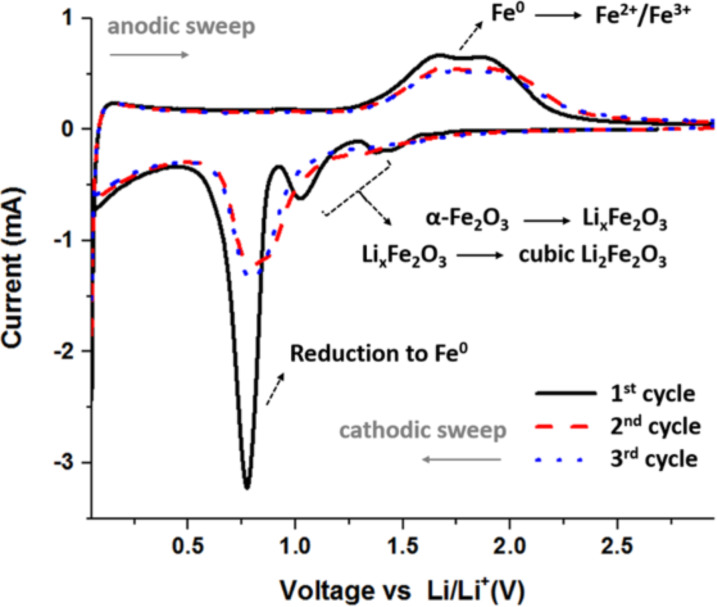
Cyclic voltamogramm profile of nanorod-shaped α-Fe_2_O_3_ (obtained with a 1.5 M Fe^3+^ concentration and ethylenediamine as the SCA).

To investigate the reaction mechanism of nanometer-scale α-Fe_2_O_3_-SX.X with lithium ions, cycling voltammetry (CV) tests were applied. The obtained data for all samples is shown in Figure 7 where the cyclic voltammetry curve for the first three cycles of α-Fe_2_O_3_-E1.5 in the voltage range of 3.00 to 0.05 V at a scan rate of 0.1 mV s^−1^ is given.

**Figure 8:**
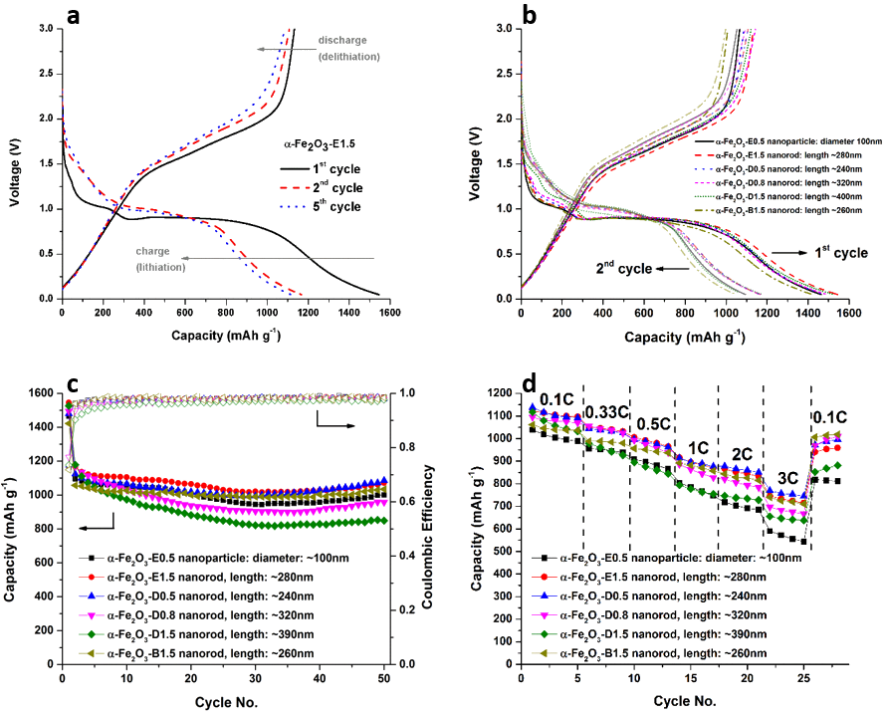
a) Galvanostatic delithiation/lithiation profiles (1st, 2nd and 5th cycle) for α-Fe_2_O_3_-E1.5, b) 1st and 2nd cycle comparison, c) cycling performance, and d) rate testing of α-Fe_2_O_3_-E0.5, α-Fe_2_O_3_-E1.5, α-Fe_2_O_3_-D0.5, α-Fe_2_O_3_-D0.8, α-Fe_2_O_3_-D1.5 and α-Fe_2_O_3_-B1.5.

For the first cathodic sweep, peaks located at 1.5 to 1.3 V and 1.1 V are observed, which correspond to lithium-ion intercalation into the α-Fe_2_O_3_ matrix with a multistep electrochemical reaction associated with the phase transformation from Li*_x_*Fe_2_O_3_ to cubic Li_2_Fe_2_O_3_ [38]. Subsequently, an intense peak at 0.78 V is presented, indicating the reduction to Fe^0^, associated with the conversion reaction leading to metallic particles finely dispersed in Li_2_O and electrolyte decomposition with solid–electrolyte interface (SEI) formation [39]. From the second cathodic cycle onwards, the peaks from 1.5 to 1.3 V and at 1.1 V do not appear and also the peak at 0.78 V shifts slightly into the higher voltage range with decreased intensity, most likely due to the irreversibility of the redox reaction as stated in the literature [14]. For the first anodic sweep, broad oxidation peaks at 1.6 and 1.8 V are observed, corresponding to the oxidation of Fe^0^ to Fe^2+^/Fe^3+^ [40]. The galvanostatic delithiation/lithiation profiles of α-Fe_2_O_3_ with nanorod morphology at 0.1 C between the voltage window of 0.05 to 3.00 V are shown in Figure 8a,b.

The obtained data are in good agreement with the CV profiles. In general, all α-Fe_2_O_3_ samples exhibit two slopes for the first charge curve from the starting voltage to around 1.2 V and from around 1.1 V to 0.8 V, which corresponds to the intercalation of lithium into α-Fe_2_O_3_, resulting in Li*_x_*Fe_2_O_3_ and cubic Li_2_Fe_2_O_3_, respectively [11,37]. A strong, pronounced plateau at around 0.8 V indicates the reduction of Fe^2+^ to Fe^0^ and formation of amorphous Li_2_O [14]. As illustrated for α-Fe_2_O_3_-E1.5 (Figure 8a), all samples exhibit a significant capacity loss during the first lithiation cycle. The first lithiation capacities for α-Fe_2_O_3_-E0.5, α-Fe_2_O_3_-E1.5, α-Fe_2_O_3_-D0.5, α-Fe_2_O_3_-D0.8, α-Fe_2_O_3_-D1.5 and α-Fe_2_O_3_-B1.5 are around 1400–1500 mAh g^−1^, and the capacity of the 1st delithiation cycle is about 1100 mAh g^−1^ with the respective coulombic efficiency of about 75%. The exact values are stated in Table 2. The initial capacity loss is possibly attributed to an incomplete conversion reaction and irreversible loss of lithium due to SEI formation [41]. The average 1st cycle lithiation voltages are calculated in the range of 0.69 to 0.75 V, while the average 1st cycle delithiation voltages are calculated from 1.43 to 1.49 V. Details for the delithiation capacity, relative capacity, error bars for cycling performance, and rate capability of α-Fe_2_O_3_-E0.5, α-Fe_2_O_3_-E1.5, α-Fe_2_O_3_-D0.5, α-Fe_2_O_3_-D0.8, α-Fe_2_O_3_-D1.5 and α-Fe_2_O_3_-B1.5 can be found in Supporting Information File 1.

**Table 2:** Electrochemical data of α-Fe_2_O_3_ synthesized with different SCAs and various Fe^3+^ concentrations.

Sample ID	1st cycle lithiation capacity (mAh g^−1^)	1st cycle delithiation capacity (mAh g^−1^)	1st cycle columbic efficiency (%)	1st cycle average lithiation voltage (V)	1st cycle average delithiation voltage (V)	1st cycle ∆*E* (V)	50th cycle lithiation capacity (mAh g^−1^)	50th cycle delithiation capacity (mAh g^−1^)

α-Fe_2_O_3_-E0.5	1465	1066	73	0.739	1.432	0.693	1001	986
α-Fe_2_O_3_-E1.5	1545	1132	73	0.746	1.436	0.690	1072	1053
α-Fe_2_O_3_-D0.5	1479	1087	73	0.745	1.433	0.688	1086	1062
α-Fe_2_O_3_-D0.8	1494	1143	77	0.743	1.494	0.751	957	935
α-Fe_2_O_3_-D1.5	1526	1121	73	0.760	1.491	0.731	848	829
α-Fe_2_O_3_-B1.5	1422	1020	72	0.726	1.447	0.721	1034	1017

The cycling performance of α-Fe_2_O_3_-E0.5, α-Fe_2_O_3_-E1.5, α-Fe_2_O_3_-D0.5, α-Fe_2_O_3_-D0.8, α-Fe_2_O_3_-D1.5 and α-Fe_2_O_3_-B1.5 at 0.1 C is illustrated in Figure 8c. Exceptional cycling stability at around 1000 mAh g^−1^ for 50 cycles was observed. In particular, samples with intermediate rod lengths (α-Fe_2_O_3_-E1.5, α-Fe_2_O_3_-D0.5 and α-Fe_2_O_3_-B1.5) in the range of ≈240 nm up to ≈280 nm show a higher capacity over spherical α-Fe_2_O_3_ nanoparticles (α-Fe_2_O_3_-E0.5) or samples with longer rod lengths of approximately ≈320 nm (α-Fe_2_O_3_-D0.8) and ≈390 nm (α-Fe_2_O_3_-D1.5). In general, the cycling behavior of the presented nanometer-scale α-Fe_2_O_3_ samples is improved over their micrometer-sized counterparts, whose capacity is reported to decrease below 200 mAh g^−1^ after a few cycles [17]. This can be attributed to the specific nanometer-scale dimensions of the material, which have several impacts: (1) improved ability to accommodate the strain during volume changes caused by lithiation/delithiation. (2) The rate of lithium insertion and removal is increased. The time constant *t* = *L*^2^/*D* (*L*: diffusion length, *D*: diffusion constant) is minimized by reducing the diffusion length *L* of the nanostructures [42]. (3) The extraction of lithium from Li_2_O becomes thermodynamically possible for nanometer-sized materials [18].

All nanometer-scale designed α-Fe_2_O_3_ samples exhibit capacity values that are far higher than those of commercialized graphite anode material at low rates (372 mAh g^−1^ for stoichiometry of LiC_6_) even in the higher C regions [8]. The samples α-Fe_2_O_3_-E1.5, α-Fe_2_O_3_-D0.5 and α-Fe_2_O_3_-B1.5 with intermediate nanorod lengths of ≈280, ≈240 and ≈260 nm show charge capacities of 746, 770 and 740 mAh g^−1^ at a charge current density of 3 C. This exhibits the superior electrode kinetics over nanoparticles (590 mAh g^−1^) and longer rods with ≈320 nm (696 mAh g^−1^) and ≈390 nm (655 mAh g^−1^) lengths. The sample α-Fe_2_O_3_-B1.5, synthesized using 2,3-diaminobutane, exhibited a nanorod length of ≈260 nm with an aspect ratio of ≈2.8 and showed the highest ability to recover the initial capacity range by providing 1018 mAh g^−1^ when the current rate was returned to its starting value of 0.1 C (Figure 8d).

A comparison of the presented α-Fe_2_O_3_ nanorods with other elongated α-Fe_2_O_3_ nanoparticles (e.g. nanorods with hexagonal structure, electrospun nanorods, and nanowires) or other morphologies (e.g. nanotubes, flower-like nanostructure, spindles, and hollow spheres) showed a notable electrochemical performance in the interaction with lithium. The results are also comparable to other iron oxides or iron oxide–carbon composites, such as Fe_3_O_4_, Fe_2_O_3_/C or Fe_3_O_4_/C, which can be also obtained by facile synthesis approaches (Table 3).

**Table 3:** Electrochemical performance of several α-Fe_2_O_3_ nanoparticles with different morphologies.

Active material	Particle size	Surface area (m^2^ g^−1^)	Current rate	Initial capacity (mAh g^−1^)	Reversible capacity (mAh g^−1^)/Cycle no.	Ref.

α-Fe_2_O_3_ nanorods	length: ≈276 nm	not specified	0.1 C	1545 (discharge)	1072/50	this work
α-Fe_2_O_3_ nanorods with hexagonal structure	length: ≈400 nm diameter: ≈40 nm	not specified	0.2 C	908 (charge)	900/30	[17]
electrospun α-Fe_2_O_3_ nanorods (sample includes 8% γ-Fe_2_O_3_)	average diameter: 150 nm	27.6 (±0.2)	50 mA g^−1^	1515 (±20) (discharge)	1095/50	[31]
α-Fe_2_O_3_ nanotubes	length: 60 µm	45	100 mA g^−1^	1415 (discharge)	510/100	[43]
α-Fe_2_O_3_ nanowires	width: ≈200 nm length/diameter ratio: 500	152	0.1 C	1303 (discharge)	456/100	[44]
α-Fe_2_O_3_ flower-like nanostructures	1–2 µm (composed of ≈20 nm thin petals)	71.9	0.2 mA cm^−2^	974.43 (discharge)	548.47/30	[45]
spindle-like mesoporous α-Fe_2_O_3_	length: ≈0.8 μm width: ≈0.4 μm, composed of clustered Fe_2_O_3_ nanoparticles <20 nm	75	0.2 C	1372 (discharge)	911/50	[46]
α-Fe_2_O_3_ hollow spheres	1 µm spherical particles composed of nanosheet subunits	103.3	200 mA g^−1^	1219 (discharge)	710/100	[47]
Fe_2_O_3_/graphene composite	carbon content: ≈20% diameter: 50–200 nm anchored on graphene	77	100 mA g^−1^	1515 (discharge)	995/50	[48]
carbon coated Fe_3_O_4_ nanospindle	carbon content: 21.5% spindle particle with length: ≈400 nm diameter: ≈100 nm	35.1	0.5 C	749 (charge, at C/5)	530/80	[49]
Fe_3_O_4_ submicron particle	diameter: 200–300 nm	not specified	0.2 C	1332 (discharge)	900/60	[50]
carbon-encapsulated Fe_3_O_4_ nanocomposite	carbon content: 30%, diameter: ≈30 nm for Fe_3_O_4_ encapsulated in carbon	110.6	93 mA g^−1^	1480 (discharge)	920/50	[51]
Fe_3_O_4_ anchored onto helical carbon nanofibers	carbon content: 50%, diameter: 10–50 nm for Fe_3_O_4_, anchored onto helical carbon nanofibers	126	80 mA g^−1^	not specified	1220/20 (fully recovered at 85 cycles after higher rate testing)	[52]
Fe_3_O_4_/graphene oxide composites	carbon content: 19% diameter 500 nm in graphene sheet	57.1	100 mA g^−1^	1233	1039/170	[53]

It should be mentioned that capacity profiles for all samples show an increase after a minimum at ≈25 cycles (Figure 8c). Especially for the shorter α-Fe_2_O_3_ nanorods (≈240 nm up to ≈280 nm) the capacity even reaches values beyond the theoretical value of 1007 mAh g^−1^ (based on the classical conversion reaction) over the monitored area. Such additional capacity occurrences and/or capacity increases in the reversible capacity regime were reported for several iron-oxide-based electrodes, including porous α-Fe_2_O_3_ nanorods [13], macroporous α-Fe_2_O_3_ submicrometer spheres [54], and 1D hollow α-Fe_2_O_3_ electrospun nanofibers [55], however its origin remains speculative. Several hypotheses are stated in the literature, among others, such as an interfacial lithium accommodation along with charge separation at phase boundaries proposed by Jamnik and Maier [56]. After conversion of α-Fe_2_O_3_, resulting in Fe finely dispersed in a Li_2_O matrix, additional lithium is supposed to be accommodated on the Li_2_O surfaces, whereas the electrons are transferred to the metal surface [57]. This mechanism strongly depends on the particle size and becomes significant in the nanometer regime [56]. Further explanations include processes related to the interfacial film formation, for instance, the possible gradual release of excessive Li^+^, previously captured by an initially inhomogeneous SEI that had sequentially developed and evolved towards a steady state over repetitive charge/discharge [58].

## Conclusion

In this work, the shape-controlling effect of various vicinal diamine derivatives, including ethylenediamine, 1,2-diaminopropane, 2,3-diaminobutane, and *N*-methylethylenediamine, towards nanorod formation of crystalline α-Fe_2_O_3_ under hydrothermal conditions was systematically studied. The position and amount of substituents (H, H_3_C) show mutual effects on the structure-directing complex formation with the ferric species (solvated, and on the surface of α-Fe_2_O_3_) due to electron density enhancement (+M effect) and steric hindrance. The shape-controlling ability was found to decrease according to: 1,2-diaminopropane > ethylenediamine ≥ 2,3-diaminobutane >> *N*-methylethylenediamine. H_3_C groups, directly located at the nitrogen (*N*-methylethylenediamine), inhibit the ferric complex formation due to steric hindrance, and therefore, the directional dependence of the crystal growth is minimized. This confirms the assumption that the structure-directing process involves the direct interaction of the free vicinal diamine unit with the ferric species. Superior shape-controlling ability is presented for 1,2-diaminopropane, resulting in nanorod formation with ≈390 nm in length and aspect ratios of ≈5.0 (1.5 M Fe^3+^ concentration), indicating that one H_3_C-group located at the same C as an amine group supports chelating of ferric species by increased electron density (+M effect) but provides less steric hindrance. However, electrochemical investigation of α-Fe_2_O_3_ nanorods as anode materials in LIBs suggests that intermediate rod lengths in the range of 240 to 280 nm show superior electrochemical performance over longer rods and in addition to spherical particles or commercial powders. This suggests that the mechanical strength against deformation during lithiation and delithiation seems to become lower for a more elongated rod shape. The highest electrochemical performance within the current test series was achieved by the samples synthesized with 1,2-diaminopropane/0.5 M Fe^3+^ (α-Fe_2_O_3_-D0.5), resulting in a nanorod length and aspect ratio of ≈240 nm and ≈2.6, respectively. The obtained reversible capacities at 0.1 C were 1086 mAh g^−1^ for α-Fe_2_O_3_-D0.5. At 50 cycles and also at a higher current rate of 3 C, this sample showed a high capacity of 1062 mAh g^−1^ and 770 mAh g^−1^, respectively, suggesting that it is a promising material that should be considered for further energy storage R&D applications.

## Supporting Information

After-cycling FESEM images for intermediate-sized and elongated nanorods, galvanostatic delithiation/lithiation and rate testing with error bars, ex situ XRD reflections for α-Fe_2_O_3_-E1.5 before and after cycling, and galvanostatic delithiation/lithiation for a half-cell composed of pure carbon black and PVdF (80% carbon black), cycled at 0.1 C.

File 1 Additional experimental results.
