# Supplementary material for: Systematic control of α-Fe2O3 crystal growth direction for improved electrochemical performance of lithium-ion battery anodes
Source: Beilstein J Nanotechnol. 2017 Sep 28;8:2032–44. doi: 10.3762/bjnano.8.204 (PMC5629383; doi:10.3762/bjnano.8.204)
Supplement: File 1 — Additional experimental results. [file Beilstein_J_Nanotechnol-08-2032-s001.pdf]

Supporting Information

for

Systematic control of α-Fe_2O_3 crystal growth direction for improved electrochemical performance of lithium-ion battery anodes

Nan Shen[1], Miriam Keppeler[1], Barbara Stiaszny[2], Holger Hain[2], Filippo Maglia[2], and Madhavi Srinivasan[1,*]

Address: [1]BMW-NTU Future Mobility Research Lab, Nanyang Technological University, School of Materials Science and Engineering, and Energy Research Institute at Nanyang (ERI@N), Research Techno Plaza, X-Frontier Blk, 50 Nanyang Drive, Singapore 637553, Singapore, and [2]BMW Group, Petuelring 130, 80788 Munich, Germany

* Corresponding author

Email: Madhavi Srinivasan - Madhavi@ntu.edu.sg

Additional experimental results

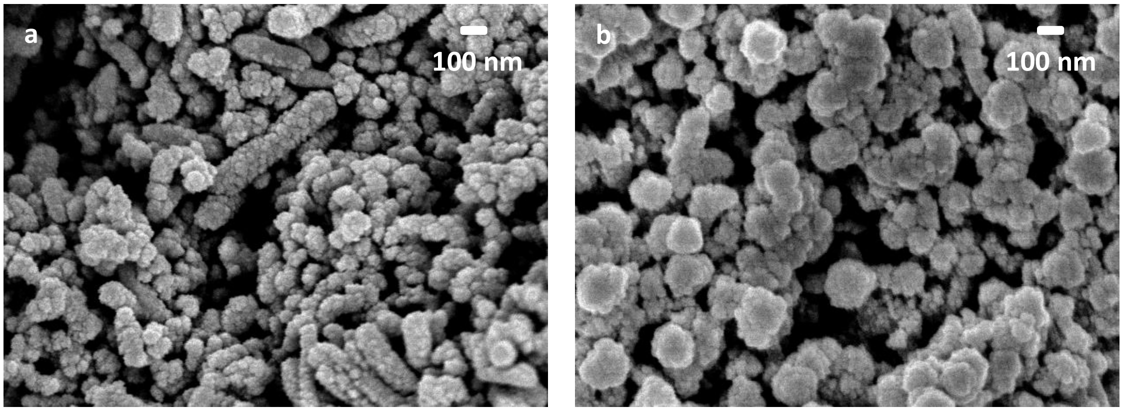

Figure S1: After-cycling FESEM images for a) intermediated sized nanorods (α-Fe_2O_3-E1.5) and b) elongated nanorods (α-Fe_2O_3-D1.5).

Coin cells (α-Fe_2O_3-E1.5, rod length ~280 nm and α-Fe_2O_3-D1.5, rod length ~390 nm) were opened after 50 cycles at 0.1 C. The electrodes were collected, and after-cycling morphology was examined by FESEM (Fig. S1). The rodlike morphology is not preserved during lithium intake and release for elongated nanorods (~390 nm), while intermediate sized nanorods (~280 nm) show a higher ability to preserve their longish shape, although the surface morphology is distorted. This observation further confirms the proposed assumption that intermediate sized α-Fe_2O_3 nanorods exhibit higher resistance to volumetric expansion during galvanostatic cycling.

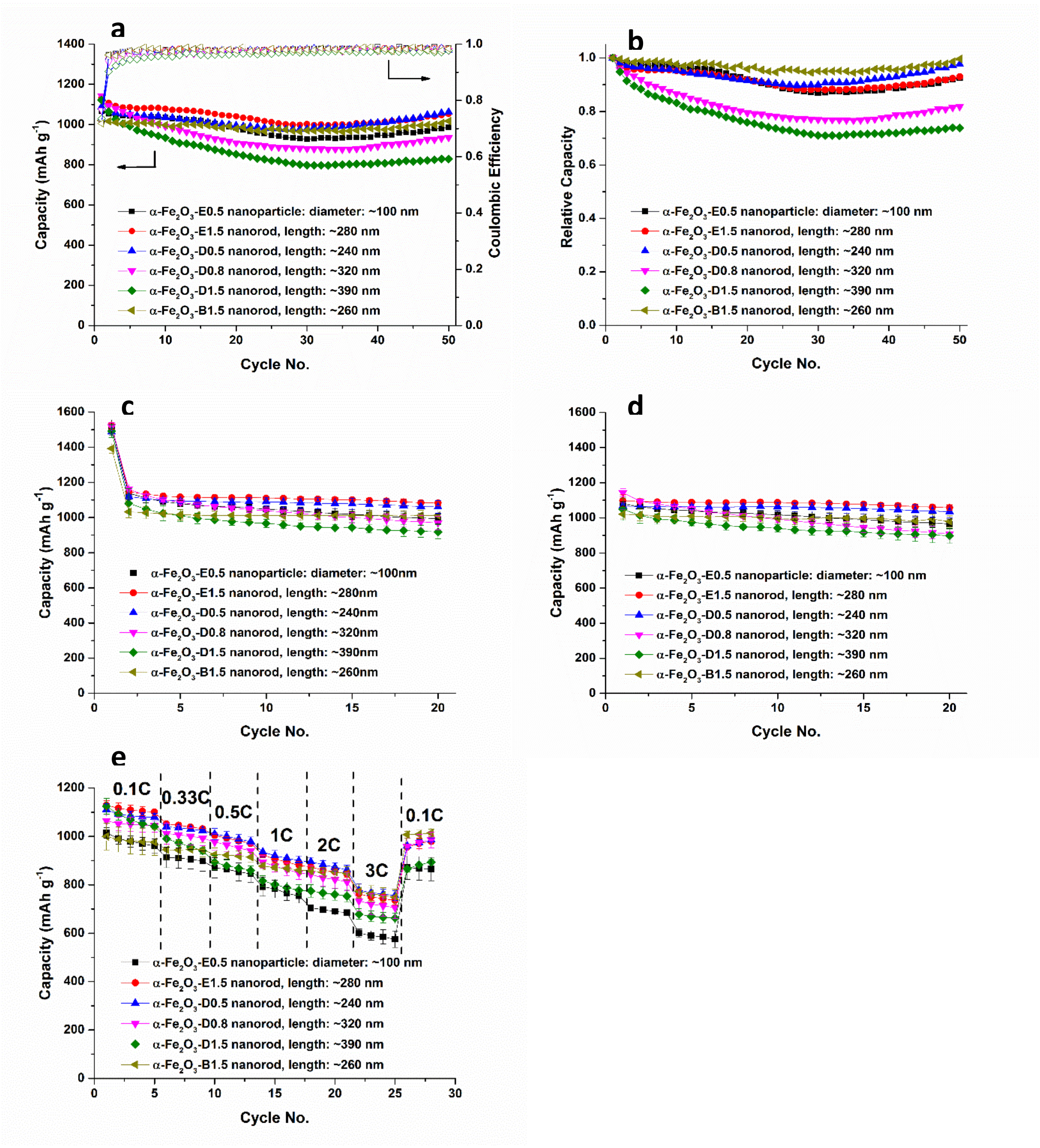

Figure S2: a) Delithiation capacity, b) relative delithiation capacity, c) error bars of lithiation capacity, d) error bars of delithiation capacity, and e) error bars of delithiation capacity for rate testing of α-Fe_2O_3-E0.5, α-Fe_2O_3-E1.5, α-Fe_2O_3-D0.5, α-Fe_2O_3-D0.8, α-Fe_2O_3-D1.5 and α-Fe_2O_3-B1.5.

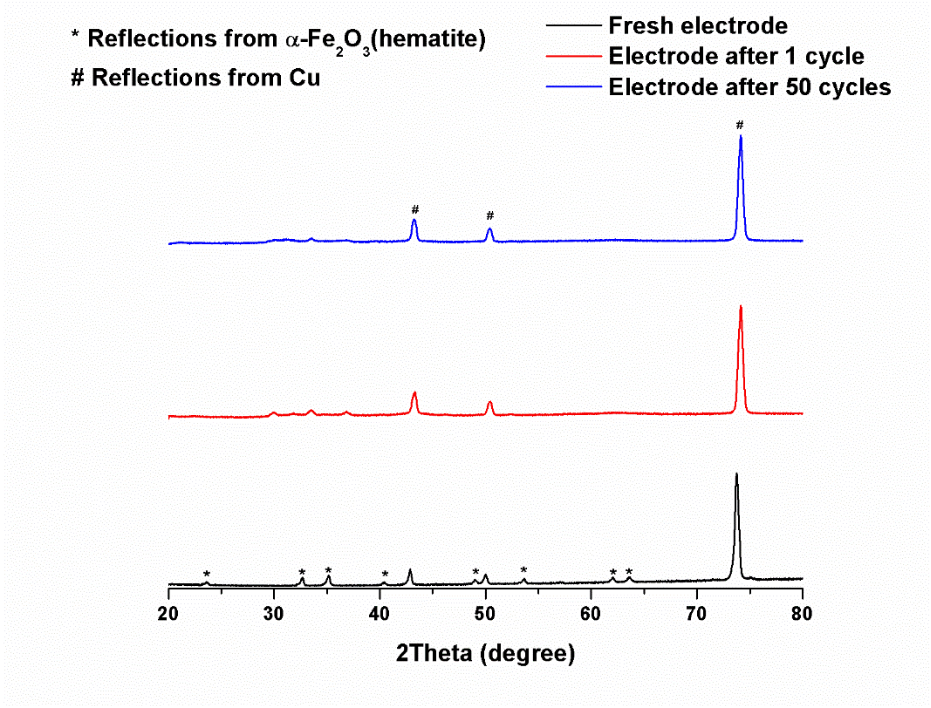

Figure S3: Ex-situ XRD reflections for α-Fe_2O_3-E1.5 before and after cycling.

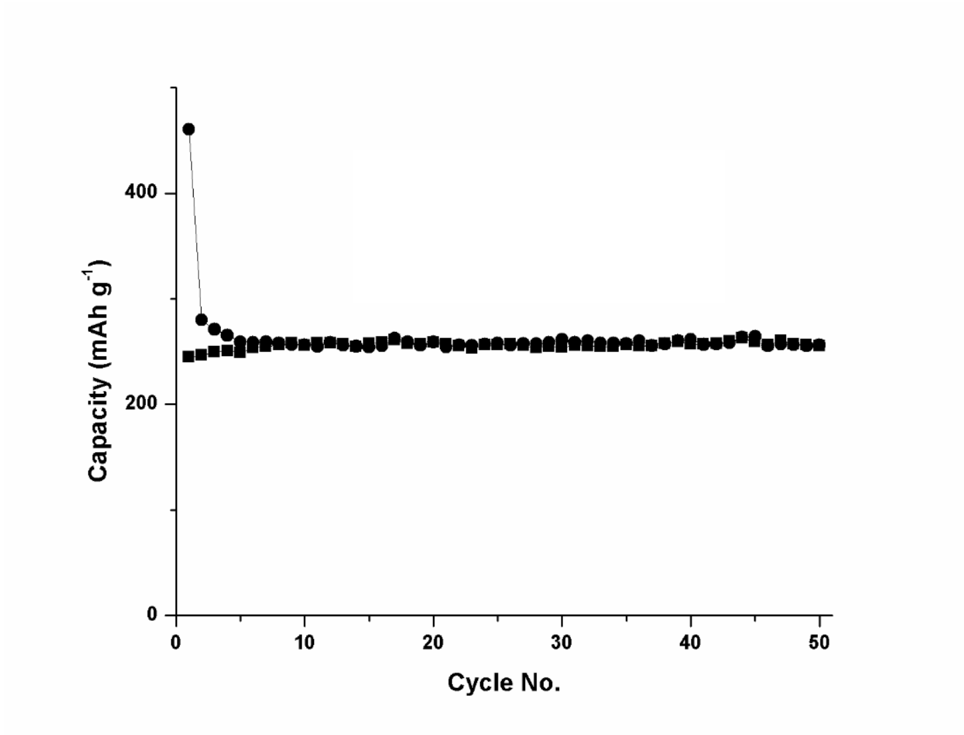

Figure S4: Half-cell composed of pure carbon black and pvdf (80% carbon black), cycled at 0.1 C.
